# Respiratory Syncytial Virus Pneumonia Treated with Lower-Dose Palivizumab in a Heart Transplant Recipient

**DOI:** 10.1155/2012/723407

**Published:** 2011-10-27

**Authors:** J. L. Grodin, K. S. Wu, E. E. Kitchell, J. Le, J. D. Mishkin, M. H. Drazner, D. W. Markham

**Affiliations:** ^1^Department of Internal Medicine, UT Southwestern Medical Center, Dallas, TX 75390, USA; ^2^Division of Infectious Diseases, Department of Internal Medicine, UT Southwestern Medical Center, Dallas, TX 75390, USA; ^3^Division of Cardiology, Department of Internal Medicine, UT Southwestern Medical Center, Dallas, TX 75390, USA

## Abstract

Respiratory syncytial virus (RSV) is an important community-acquired pathogen that can cause significant morbidity and mortality in patients who have compromised pulmonary function, are elderly, or are immunosuppressed. This paper describes a 70-year-old man with a remote history of heart transplantation who presented with signs and symptoms of pneumonia. Chest computed tomography (CT) imaging demonstrated new patchy ground glass infiltrates throughout the upper and lower lobes of the left lung, and the RSV direct fluorescence antibody (DFA) was positive. The patient received aerosolized ribavirin, one dose of intravenous immunoglobulin, and one dose of palivizumab. After two months of followup, the patient had improved infiltrates on chest CT, improved pulmonary function testing, and no evidence of graft rejection or dysfunction. There are few data on RSV infections in heart transplant patients, but this case highlights the importance of considering this potentially serious infection and introduces a novel method of treatment.

## 1. Introduction

Respiratory syncytial virus (RSV) is an enveloped RNA virus belonging to the family of Paramyxoviridae and is primarily known as a common infection in children [1]. However, this community-acquired respiratory viral infection can cause significant morbidity and mortality in patients who have compromised pulmonary function, are elderly, or are immunosuppressed [2, 3]. 

 Other respiratory viruses have been reported in adult heart transplant recipients [4–6]. There are little data regarding the natural history, treatment, and prognosis of RSV pneumonia in adult heart transplant recipients. We reviewed two previous case reports in the literature [7, 8]. The first report described a patient who became ill 60 days post-transplantation and was treated successfully with aerosolized ribavirin. The second describes a patient with a fever of unknown origin who was diagnosed with RSV pneumonia by gallium scan three years posttransplantation. In this paper there is no discussion of treatment or outcome. Our patient is unique as he was 10 years posttransplant and had been weaned off prednisone.

## 2. Case Report

**Figure 1:**
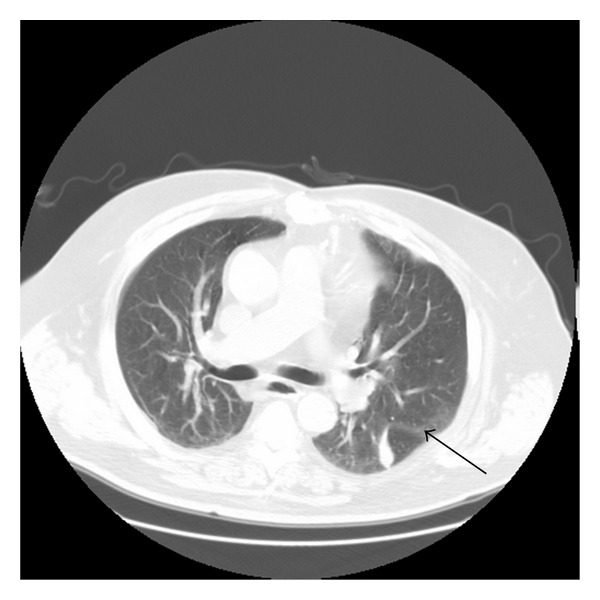
Chest computed tomogram with intravenous contrast two months prior to admission revealing atelectasis of the left lower lobe (arrow).

**Figure 2:**
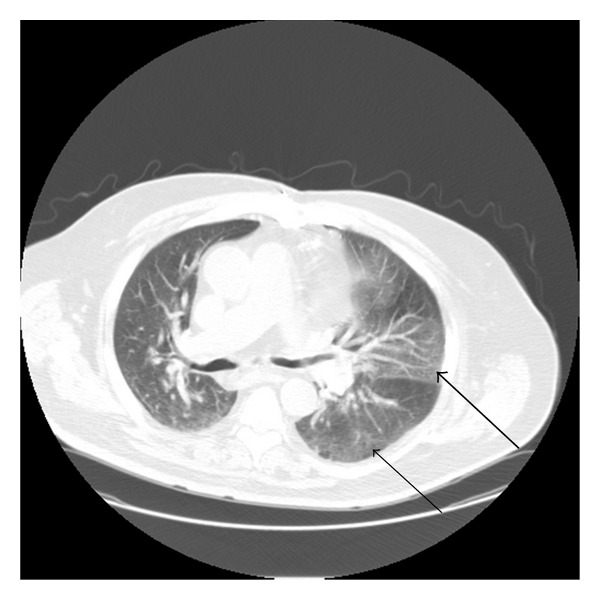
Admission chest computed tomogram with intravenous contrast revealing new ground glass infiltrates of the upper (bold arrow) and lower (narrow arrow) lobes of the left lung.

A 70-year-old Caucasian man with a history of orthotopic heart transplantation (OHT) in 1999 for an ischemic cardiomyopathy presented with a five-day history of rhinorrhea, mylagias, and subjective fevers. In addition, he noted worsening orthopnea and an inability to lay flat without using a continuous positive airway pressure device. Subsequently he developed dyspnea with a productive cough of gray/white sputum. A routine posttransplant computed tomogram (CT) scan of the chest six months prior to admission revealed extensive atelectasis in the basal segments of the left lower lobe and lingula. A repeat CT scan two months prior to admission revealed worsening atelectasis of the left lower lobe (Figure 1). He therefore underwent a transbronchial biopsy of his left lower lobe. The pathology was unremarkable. Bronchial fluid cultures grew normal respiratory flora and cytology was negative for malignancy. Pulmonary function tests (PFTs) at that time were consistent with a restrictive physiology (Table 1). On the day of admission, the patient presented with symptoms suggestive of an acute infectious upper and lower respiratory process and worsening orthopnea from baseline. The chest radiograph (not shown) and CT of his chest at this time revealed new patchy ground glass infiltrates throughout the upper and lower lobes of the left lung (Figure 2).

Past medical history includes Type II diabetes mellitus with nephropathy, retinopathy, and neuropathy; obesity (BMI of 39.5 kg/m^2^); nonalcoholic fatty liver disease; chronic peripheral edema and venous stasis; resected melanoma; basal cell carcinoma of the face; hypertension; dyslipidemia. He was a former heavy smoker prior to his heart transplantation, rarely consumed alcoholic beverages, denied any previous illicit or intravenous drug abuse, and was unable to recall any recent sick contacts. Medications on admission were cyclosporine (100 mg orally twice daily), mycophenolate mofetil (250 mg orally twice daily), insulin glargine, furosemide, simvastatin, enalapril, trimethoprim-sulfamethoxazole, diltiazem, acetaminophen, aspirin, fenofibrate, vitamin E, and vitamin C.

 Physical examination revealed an obese man in mild respiratory distress. He was afebrile with a temperature of 36.7 degrees Celsius. The pulse was 118 and regular, blood pressure 148/74, oxygen saturation 88% on room air improving to 94% on 3 liters of oxygen, and weight 118 kg. He was breathing approximately 24 times per minute and had bilateral rales at the bases of his lungs. Other than tachycardia, his cardiovascular exam was unremarkable. The remainder of his examination was remarkable for 3+ pitting edema of the lower extremities with venous stasis changes up to the superior portion of his calves.

 Upon arrival to the intensive care unit, an arterial blood gas revealed a pH of 7.29, PCO_2_ of 74 mmHg, and PO_2_ of 48 mmHg. The results of multiple serologies and cultures were negative except for a positive RSV direct fluorescence antibody (DFA).

**Figure 3:**
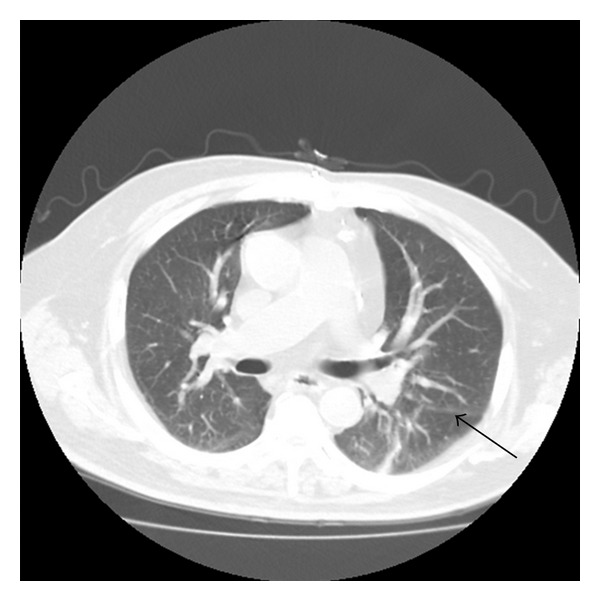
Chest computed tomogram with intravenous contrast three months after admission with resolved ground glass infiltrates revealing the initial atelectasis of the left lower lobe present two months prior to admission (arrow).

 Given the severity of his symptoms, he was treated with 5 days of aerosolized ribavirin (Virazole 2 gm inhaled every 8 hours), one dose of IV immunoglobulin (Gamunex 400 mg/kg = 50,000 mg), and, due to financial constraints, a single half-dose of palivizumab (Synagis 7.5 mg/kg = 900 mg) [9]. In addition, he received temporary bilevel positive airway pressure ventilation (BiPAP) and 3 days of intravenous methylprednisolone (40 mg daily). After five days of treatment, a repeat RSV DFA was negative. He was subsequently weaned off of BiPAP and released from the hospital. Three months later, a follow-up CT scan of his chest revealed resolution of his ground glass infiltrates (Figure 3) and improvement of his PFTs from baseline (Table 1). He had no evidence of allograft rejection or dysfunction.

## 3. Discussion

In the United States, most RSV infections occur throughout a period of approximately 22 weeks from the late fall to late spring [1]. Peak activity occurs during the months of January and February. Syndromes of RSV infection in adults include mild upper respiratory illnesses, bronchitis/bronchiolitis, pneumonia, and fulminant respiratory failure [3].

We find this paper clinically essential as there is both a paucity of information regarding the treatment for RSV pneumonia in OHT recipients and a lower dose of palivizumab may be sufficient. We treated our patient based on the experience reported in the bone marrow and lung transplant literature [6–8]. Aerosolized ribavirin decreases viral load in adults following bone marrow transplantation but may have little efficacy on mortality [10]. In patients with lung transplantation, aerosolized ribavirin increases the likelihood of improvement in forced expiratory volume in one second, lowers rates of bronchiolitis obliterans, and may yield a complete recovery [10–12]. Evidence suggests that the addition of palivizumab (a humanized anti-RSV antibody) or intravenous immunoglobulin (IVIG) may improve survival in patients after bone marrow transplantation and decrease the incidence of bronchiolitis obliterans and rejection in patients after lung transplantation [13–16]. However, at the recommended dose (Table 2) [9], palivizumab treatment in an adult may cost more than $10,000 [17]. Newer experimental therapies have been reviewed elsewhere [18]. To date there is no consensus on treatment of RSV pneumonia, but it has been suggested that use of aerosolized ribavirin and antibody therapy should be considered in patients with upper respiratory tract symptoms and other risk factors (i.e., posttransplant immunosuppression) [19].

## 4. Conclusion

In summary, little is known about RSV infections in orthotopic heart transplant recipients. Given the serious consequences of RSV pneumonia following bone marrow or lung transplantation, RSV pneumonia should be included in the differential diagnosis in patients after OHT who present with a viral prodrome and abnormal lung imaging. Although there are no data regarding treatment and outcomes of RSV infection in heart transplant recipients, our patient appeared to have good clinical response to an approach from the bone marrow and lung transplant literature with a lower palivizumab dose. Further study regarding the frequency and optimal therapeutic approach for RSV infections in heart transplant recipients is needed.

##  Conflict of Interests

The authors of this paper have no conflict of interests to disclose.

##  Authors' Contribution

All authors contributed in drafting, critical revision, and approval of this paper.

## Figures and Tables

**Table 1:** Pulmonary function tests two months prior to admission and at three months followup.

PFT	Prior to admission	Followup
FVC (liters), (% predicted)	2.01, (45%)	2.76, (68%)
FEV1 (liters), (% predicted)	1.58, (53%)	2.21, (74%)
FEV1/FVC (% predicted)	78	80

FVC: forced vital capacity, FEV1: forced expiratory volume in one second.

**Table 2:** Common treatments for RSV respiratory tract infection.

Drug	Dosage	Frequency
Ribavirin		
Aerosolized	2 g	Every 8 hours for 15 total doses
Oral / IV*	15–20 mg/kg	Divided in 3 doses over 10 days
Corticosteroids^†^		
Solu-Medrol	10–15 mg/kg/day	Over 3 days
IVIG	0.5 g/kg	Once
Palivizumab	15 mg/kg	Once
Motavizumab^‡^	3–15 mg/kg	Once

RSV: respiratory syncytial virus, IVIG: intravenous immunoglobulin.

*IV ribavirin with oral corticosteroids is well tolerated and effective with a lower price than aerosolized ribavirin [20].

^†^There is no consensus as to the appropriate regimen. Steroid selection and dosing are usually center dependent.

^‡^Motavizumab is a new, potent anti-RSV immunoglobulin, recently not approved by the FDA in a recent filing for licensure [21].
